# Cost-Effective Strategies for Rural Community Outreach, Hawaii, 2010–2011

**DOI:** 10.5888/pcd11.140315

**Published:** 2014-12-11

**Authors:** Karen L. Pellegrin, Anna Barbato, R. Scott Holuby, Anita E. Ciarleglio, Ronald Taniguchi

**Affiliations:** Author Affiliations: Anna Barbato, Anita E. Ciarleglio, Ronald Taniguchi, University of Hawaii at Hilo, Hilo, Hawaii; R. Scott Holuby, San Antonio Military Medical Center, San Antonio, Texas.

## Abstract

Three strategies designed to maximize attendance at educational sessions on chronic disease medication safety in older adults in rural areas were implemented sequentially and compared for cost-effectiveness: 1) existing community groups and events, 2) formal advertisement, and 3) employer-based outreach. Cost-effectiveness was measured by comparing overall cost per attendee recruited and number of attendees per event. The overall cost per attendee was substantially higher for the formal advertising strategy, which produced the lowest number of attendees per event. Leveraging existing community events and employers in rural areas was more cost-effective than formal advertisement for recruiting rural community members.

## Objective

Medication safety is an important educational topic; adverse drug reactions in older adults are a common cause of hospital admissions, illness, and death (1,2). Adults aged 65 or older have relatively high rates of preventable adverse drug reactions because of their complex drug regimens for chronic disease and sensitivity to commonly used drugs (3,4). These factors frequently result in emergency hospitalizations of older adults (2). Group-based health education can be both effective and efficient in preventing such outcomes and may be particularly beneficial in rural areas where shortages of health care professionals are common (5,6). The objective of this study was to evaluate the cost-effectiveness of recruitment strategies designed to maximize attendance at medication safety training throughout rural communities in Hawaii.

## Methods

This study used a quasi-experimental design to compare the cost-effectiveness of 3 recruitment strategies designed and implemented sequentially during a 14-month period of 2010–2011 to maximize attendance at education sessions focused on safety of medications commonly used to manage chronic disease. We targeted all counties in Hawaii classified as “non-metro” according to the US Department of Agriculture (USDA) 2003 Rural-Urban Continuum Codes (ie, Kauai, Maui, and Hawaii counties). The target audience was older adults and their caregivers. This research was approved by the institutional review board of the University of Hawaii. The strategies are described in order of implementation.

### Existing community groups and events

Key contacts with existing community groups were identified in all target counties. From July through November 2010, the education sessions were held in all 3 counties during regularly scheduled meetings or other existing events, at venues such as senior centers, neighborhood centers, churches, and community fairs. Although the sessions were typically 1 hour, a 30-minute format was used when the host specified a time limitation.

### Formal advertisement

The education sessions were scheduled on weekends (1-hour format), and advertised via local media (both print and radio) before the events. The advertisements were professionally designed and produced to maximize attendance. This approach was discontinued after implementation during March 2011 in 2 of the 3 targeted counties after preliminary results indicated it was not effective.

### Employer-based outreach

The human resources offices of larger employers in each of the target counties (as published in the *Pacific Business News* “Book of Lists”) were contacted. During July and August 2011, the educational sessions were held in all 3 counties and scheduled at times to maximize attendance, including employee breaks, shift changes, and regularly scheduled meetings of their retirees. Although the sessions were typically 1 hour, a 30-minute format was used when the host specified a time limitation.

For each strategy, the costs of outreach efforts were tracked (including staff time, consultant fees, and materials), along with attendance at each event. Relative cost-effectiveness was measured by comparing overall cost per attendee as well as number of attendees per event.

## Results

A total of 1,002 people attended the educational sessions on medication safety for older adults throughout all 3 target counties in Hawaii. We recorded the number of attendees and calculated recruitment costs for all 3 strategies (Table). The overall cost per attendee was much higher for the formal advertising strategy than for other strategies and produced the lowest number of attendees per event. The sessions coordinated through existing community groups resulted in the highest number of attendees per event with a cost per attendee that was only slightly higher than that of the employer-based sessions.

**Table:** Comparison of the Recruitment Strategies Implemented Sequentially in Rural Counties, Hawaii, 2010–2011

Strategy	No. of Events	Total No. of Participants	Total Recruiting Cost, $	No. of Participants per Event	Cost per Participant, $
Community groups/events	24	729	10,071	30	13.81
Formal advertisement	3	54	16,955	18	313.98
Employer-based outreach	11	219	2,649	20	12.10

## Discussion

Group-based approaches can be an effective way to deliver health-related education and are often selected for their efficiency (5,6). They may be particularly beneficial in rural areas where there are shortages of health care professionals. However, there is a paucity of research on recruitment strategies for participation in group-based education. The few published studies on rural recruitment for participation in research indicate that strategies leveraging relationships and existing community resources are most effective (7–11). None of these studies compared the cost-effectiveness among the approaches. To fill this research gap, we examined the cost-effectiveness of rural recruitment strategies designed to maximize attendance at education sessions on chronic disease medication safety. Consistent with the research on rural recruitment strategies for research, recruitment approaches designed to leverage existing relationships and community resources (ie, community groups, events, and employers) were more successful than traditional advertising. In addition, the advertising approach was substantially more expensive than the other approaches.

Further research is needed because this study was a quasi-experimental design with important limitations. The program was implemented only in rural communities in Hawaii and on a focused topic, which limits the ability to generalize to other communities and content. The recruitment strategies were implemented sequentially and without experimental control groups. This design weakness introduces confounders and prevents the analysis of additive and interaction effects. For example, the formal advertisement strategy was the only strategy implemented during the spring, creating a seasonal confounder. In addition, the sequencing of the strategies may have affected the results by increasing general community awareness of the sessions over time. However, the findings are consistent with those of previous studies of rural recruitment strategies, which enhances their validity.

Further research on identification of cost-effective strategies for recruiting community members to attend educational events for chronic disease management will increase the impact of such health education programs. This study, combined with previous research, suggests that recruiting rural community members to participate in health-related educational activities — whether for prevention, treatment, or clinical research — should focus on leveraging existing social networks (eg, community groups and employers) rather than formal advertisement.
